# Shining evolutionary light on human sleep and sleep disorders

**DOI:** 10.1093/emph/eow018

**Published:** 2016-07-28

**Authors:** Charles L. Nunn, David R. Samson, Andrew D. Krystal

**Affiliations:** ^1^Department of Evolutionary Anthropology, Duke University, Durham, North Carolina 27708, USA; ^2^Duke Global Health Institute, Durham, North Carolina 27710, USA; ^3^Triangle Center for Evolutionary Medicine, Durham, NC 27708, USA; ^4^Department of Psychiatry and Behavioral Sciences, Duke University School of Medicine, Durham, NC 27710

**Keywords:** sleep disorder, evolutionary mismatch, comparative study, phylogeny, human health, human evolution.

## Abstract

Sleep is essential to cognitive function and health in humans, yet the ultimate reasons for sleep—i.e. ‘why’ sleep evolved—remain mysterious. We integrate findings from human sleep studies, the ethnographic record, and the ecology and evolution of mammalian sleep to better understand sleep along the human lineage and in the modern world. Compared to other primates, sleep in great apes has undergone substantial evolutionary change, with all great apes building a sleeping platform or ‘nest’. Further evolutionary change characterizes human sleep, with humans having the shortest sleep duration, yet the highest proportion of rapid eye movement sleep among primates. These changes likely reflect that our ancestors experienced fitness benefits from being active for a greater portion of the 24-h cycle than other primates, potentially related to advantages arising from learning, socializing and defending against predators and hostile conspecifics. Perspectives from evolutionary medicine have implications for understanding sleep disorders; we consider these perspectives in the context of insomnia, narcolepsy, seasonal affective disorder, circadian rhythm disorders and sleep apnea. We also identify how human sleep today differs from sleep through most of human evolution, and the implications of these changes for global health and health disparities. More generally, our review highlights the importance of phylogenetic comparisons in understanding human health, including well-known links between sleep, cognitive performance and health in humans.

## INTRODUCTION

Sleep is essential to cognitive function and health in humans. For example, experiments have shown that sleep is important for working memory, attention, decision-making, and visual-motor performance [1–3]. Chronic sleep deprivation and alterations in circadian rhythms, such as shift work, also increase the risks for obesity, hypertension, heart disease and immune system dysfunction, which may increase the risks for infection, inflammation, and some types of cancer [4–8]. In the USA, 50–70 million Americans suffer from chronic sleep disorders, and 20% of serious automobile accidents are attributable to sleep deprivation [9]. Although less is known about global variation in sleep patterns [10], rates of sleep problems and chronic sleep deprivation are probably increasing in developing countries, where aging populations, transitions to market economies, and adoption of Western lifestyles are altering sleep patterns [11–13].

Despite growing appreciation of the importance of sleep, the ultimate reasons for sleep remain mysterious. Sleep appears to help rejuvenate the brain, including purging byproducts of metabolism that accumulate during the day [14]. The growing realization that sleep interconnects deeply with many other physiological and cognitive mechanisms suggests that sleep has many functions, including growth and repair of the body (e.g. release of growth hormone, 15), immune function [16, 17], and even adaptive stillness to avoid predation [18, 19]. These functions are likely to vary in importance across species, including in humans compared to other primates. In addition, evolutionary perspectives involving tradeoffs are important for understanding sleep, including tradeoffs between sleep and other fitness relevant activities such as foraging or caring for offspring, and also pleiotropic effects of genes on sleep and related physiological processes.

We need to understand why humans sleep the way we do, why sleep deprivation is so detrimental to our health through various neurological and physiological mechanisms, and how we can sleep better. Here, we integrate recent findings from human sleep studies and the ecology and evolution of sleep, with the goal to deepen our understanding of human sleep, including sleep disorders and the global health implications of sleep deficiency. A central premise of our article is that human sleep has undergone changes from our primate ancestors [20, 21]. These derived characteristics (and their correlates) may hold important clues to understanding the links between sleep, cognitive performance and human health. Another premise is that humans in the developed world sleep differently than our ancestors did [21, 22]. These changes partly arise through increased access to electrical lighting in the developed world, but also through our use of separate bedrooms, soft beds and cultural norms against daytime napping. A final premise is that evolutionary concepts, such as tradeoffs, are important for understanding human sleep.

We begin by considering patterns of human sleep in relation to other primates, including the ways that human sleep differs from our close evolutionary relatives. We also review recent hypotheses involving sleep and infants [23, 24]. In an effort to understand the reasons why human sleep differs from other primates, we review our knowledge of sleep patterns across mammals, focusing on the correlates of that variation. We also provide evolutionary perspectives on several major sleep disorders, and on links between poor or disrupted sleep and health disparities. We suggest that sleep deprivation is a largely unrecognized global health problem that may contribute to both infectious and non-infectious disease risks in developing countries, and to health disparities in developed countries.

## HUMAN SLEEP IN PRIMATE PERSPECTIVE

Most primate species are arboreal, and this appears to be the ancestral state for primates [25]. Kappeler [26] used primate life history traits to reconstruct the evolutionary history of sleep site usage. His analysis revealed that the ancestral primate probably resembled extant galagos: they were likely to be nocturnal, solitary and producing a single offspring that was provisioned in a tree-hole nest, or ‘fixed-point’ sleep site. A primary advantage of these fixed-point sleep sites may have been increased safety from predators [27], along with improved thermoregulation [28].

Like many other mammals, the primate lineages emanating from the Paleocene evolved increased body size [29, 30]. This increase in body size led primates on many of these lineages to abandon fixed-point sleep sites, as naturally occurring enclosed sites would be challenging for larger animals to find. Similarly, the evolution of diurnal activity patterns—and associated shifts to living in larger groups [31]—would have made it even more difficult for larger groups of animals to locate fixed point sleep sites. These factors led early primates to abandon the advantages of enclosed and sturdy sleep sites, and to instead sleep on tree branches. Sleeping on branches would have exposed these animals to increased risks from predation and to falling, especially because wind speeds, with punctuated gusts, are greater in the canopy [32]. Indeed, the primatology literature provides multiple accounts of primates falling from arboreal sleeping sites, resulting in injuries and death [33, 34].

### Great ape sleep

A major evolutionary transition in sleep likely occurred in the ancestor of the great apes: humans, orangutans, gorillas, chimpanzees and bonobos all build platforms (or ‘nests’) upon which to sleep [35–37]. Great ape sleeping platforms show a conserved pattern of construction and function, and phylogenetic reconstruction points to emergence of this sleeping behavior sometime between 18 and 14 million years ago [38]. Typically, these platforms are built in trees that are selected for their firm, stable and resilient biomechanical properties [39–41]. Platforms are rebuilt each night, with each individual (except dependent young) building a separate sleeping nest. In sharp contrast, the lesser apes—the gibbons—do not build nests for sleeping. Instead, gibbons follow the pattern found in most monkeys: they typically sleep on branches in a lying or sitting position, with no environmental alterations [42, 43].

Why do great apes build sleeping platforms? Based on evidence showing a link between sleep and cognition in humans and great apes, the ‘sleep quality hypothesis’ proposes that more stable sleeping sites provide physical support needed for large bodied hominoids to maintain deep, sustained sleep to enable enhanced cognitive function [37, 44, 45]. An alternative ‘engineering hypothesis’ states that great ape platform building simply reflects greater cognitive ability, which enables the great apes to build nests [44]. This is a simple reversal of cause and effect, where the cause is greater cognitive facility providing the opportunity to build effective sleep platforms, rather than use of platforms to enable greater cognitive performance.

Recent captive research on apes has tested two crucial elements of the sleep quality hypothesis for the use of sleeping platforms in great apes. In a zoo study, Samson and Shumaker [46] provided orangutans with varied sleep materials, and then scored the quality of sleeping platforms the orangutans produced with different materials. They found that sleeping platform quality was positively correlated with reduced arousability and lower sleep fragmentation (i.e. metrics of better quality sleep). In another study of zoo animals, Martin-Ordas and Call [47] found that, by making memory more resistance to the detrimental effects of interfering (i.e. distracting) activities, sleep plays a role in memory consolidation in chimpanzees, bonobos and orangutans.

Increased body mass likely also played a role in the origins of great ape sleeping platforms [21]. In particular, larger-bodied great apes would find it more difficult to sleep on tree branches. This effect would have favored individuals that built more resilient sleeping platforms to reduce the probability of lethal falls, and to reduce physical stressors on the bodies of sleeping individuals. A distinct mass threshold (∼30 kg) has been proposed that separates the great apes that use sleeping platforms from the lesser apes and monkeys that do not [21, 45]. Once the use of sleeping platforms evolved, this could have enabled higher quality sleep within great apes, with emergent cognitive benefits.

### Human sleep

Human sleep has undergone additional changes from other great apes in several key features. An obvious feature is where we sleep, namely on the ground; among other apes, terrestrial sleep is rare, occurring only when predation risk is low, and typically only by very large bodied males [48–51]. In contrast, humans of both sexes habitually sleep on the ground, which could plausibly provide even more stable sleeping locations to achieve even deeper sleep. Predation represents a major tradeoff in this context, with risk of predator attack thought to increase for terrestrial primates [52, 53].

In relation to human ground sleep, Coolidge and Wynn [54] proposed the ‘tree-to-ground hypothesis’. They suggested that when hominins became fully terrestrial they gained the advantage of greater stability than was possible in arboreal sleep. Freed from the disadvantages of arboreal sleep they could have achieved longer duration and higher quality sleep, which would have improved waking cognition. Without terrestrial sleeping sites, they argue, fully human procedural memory consolidation for visual-motor skills and visual-spatial locations could not have evolved. In addition, under the assumption that sleep plays a role in problem solving in social and other domains involving ‘threat simulation’ [55], they proposed that hominins would have been less primed for daily activity due to less sleep the previous night [20, 54].

The controlled use of fire may have been an essential precursor to secure ground sleep [20]. Arboreal sleeping platforms reduce predation risk [56] and minimize insect biting rates by masking host attractants or actually repelling insects [57, 58]. Sleeping platforms also provide some insulation for warmth [57], and give a stable and secure environment to enable higher quality sleep [39, 40]. A fire probably also reduces risk of predation and provides opportunities for thermoregulation, while smoke reduces insect activity [59, 60]. Control of fire in early *Homo erectus* may therefore have enabled the night-time transition from trees to the ground [20, 61].

**Figure 1.**
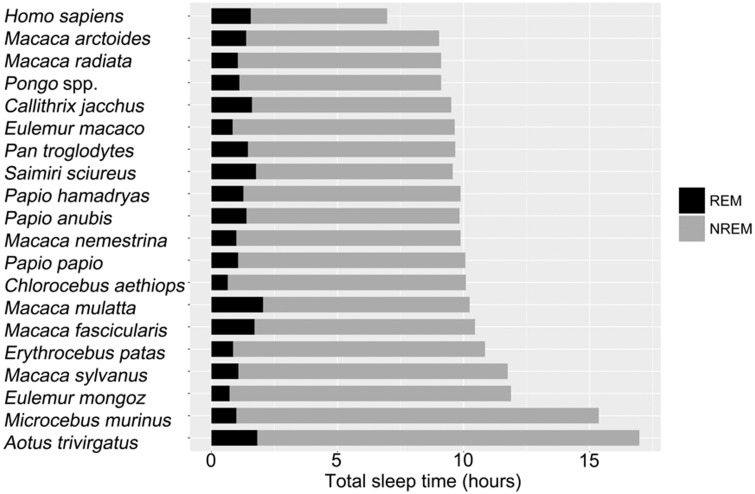
Duration of REM, NREM and total sleep in primates. Humans sleep the least compared to all other primates, yet have the greatest proportion of total sleep time dedicated to REM

Quantitative characteristics of human sleep have also evolved along the human lineage. We consider here two major aspects: reduced total sleep and a higher percentage of rapid eye movement (REM) sleep [21]. Humans are empirically the shortest sleeping primates and have the highest percentage of REM (Fig. 1). New phylogenetic methods can rigorously investigate evolutionary change on a single branch, allowing a comparative biologist to investigate whether an exceptional amount of evolutionary change has occurred [62, 63]. More specifically, these methods compare actual sleep characteristics in humans to the predicted outcomes from a statistical model that includes both phylogeny and a set of predictor variables that influence sleep characteristics. One can then test whether humans are a typical primate (our observed sleep duration falls within the predicted 95% credible interval) or a ‘phylogenetic outlier’ (our sleep duration falls outside the predicted 95% credible interval).

Using this approach, Samson and Nunn [21] discovered that human sleep duration is extremely different from phylogenetic predictions: our actual sleep duration falls outside the 95% credible interval, suggesting that we can be more than 95% certain that human sleep differs from other primates. As we discuss below when considering the potential evolutionary drivers of shorter sleep along the human lineage, tradeoffs between sleep and other activities are likely to be important factors. When this same approach was applied to study the proportion of REM sleep in humans, the analyses revealed that humans pack a higher proportion of REM into their sleep than any other primate. It is worth noting, however, that some other primates have a longer absolute duration of REM sleep (see Fig. 1).

As a last point of comparison to other primates, humans may be more flexible in the timing of sleep than our closest living relatives. Evidence from small-scale societies and subtropical hunter-gatherers [22], the historical record [64] and experiments in developed countries [65] suggest that humans show flexibility in their sleep. In a review of human sleep across cultures, Worthman [22] noted that, ‘Human nights are filled with activity and significance, and nowhere do people typically sleep from evening to dawn’ (p. 301). Similarly, reflecting on his study of the Pirahã hunter-gatherers in South America, Everett (66) noted, ‘Pirahãs take naps (fifteen minutes to two hours at the extremes) during the day and night. There is loud talking in the village all night long’ (p. 79). Similar patterns appeared to occur in European and equatorial societies prior to the advent of cheap and effective lighting, with a historical analysis documenting extensive use of the concept of ‘first sleep’ and ‘second sleep’, consistent with a biphasic sleep pattern that differs radically from what we consider ‘normal’ in Western societies today [64, 67]. Flexibility can also occur in the context of daytime sleep, i.e. the occurrence of napping or siestas. For example, Pennsylvanian Old Order Amish, a conservative Christian sect that avoids modern electrical conveniences, have been characterized as ‘common’ nap-takers, with 58% of the population recording a nap a least once per week [68].

Counter to these findings and suggestions, however, a recent study of sleep in three hunter-gatherer populations [69] interpreted their actigraphy data as indicating consolidated sleep at night and with little napping during the day, and thus arguing against the flexibility of sleep. This presents a challenge, and calls for better methods of assessing sleep phasing using actigraphy, including through use of new algorithms, validation with reported episodes of sleep and wakefulness, and development of new methods to better assess sleep without reliance on actigraphy. It should be noted, however, that this study also revealed considerable heterogeneity in sleep onset time (but less in awakening), consistent with flexibility in the timing of sleep.

Given the global distribution of humans, adaptation to local conditions may be expected for sleep, as seen for other human phenotypes. One obvious aspect of this involves latitude, and the effects of large changes in day-length throughout the year. Unfortunately, however, sleep research in circumpolar environments has primarily focused on European populations [70, 71] and the effects of latitude on the physiology of military personnel [72]. Thus, little is known regarding the effects of seasonally variable day–night cycles on the sleep-wake patterns of nonindustrial indigenous populations [12]. Moreover, reports of sleep in post-industrial societies have shown conflicting evidence and small effects with respect to sleep duration across seasons [73, 74]. Several factors may influence the outcome of such studies, including lack of direct exposure to changes in light and temperature among participants in laboratory environments, or the environmental buffer provided by modern work and residential facilities. In contrast, evidence supports the idea that sleep is modulated by season in traditional, equatorial societies; e.g. longer total sleep times (53–56 min increase) were associated with the ‘winter’ season in the San and Tsimane [69].

### Sleep and human development

**Figure 2.**
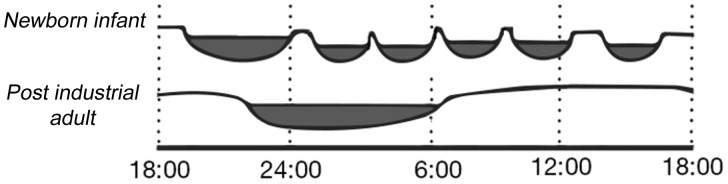
Infant versus adult sleep**.** A sleep comparison between polyphasic human infant and consolidated sleep in an adult living in a post-industrial society (adapted from reference [75])

Ontogeny can also shed light on human sleep. As all parents know, babies sleep a lot, yet they are born without a regular sleeping rhythm (Fig. 2). The chaos of sleep phasing in the first days of life consolidates into a polyphasic sleep schedule consisting of at first two naps and one bout of night-time sleep, and eventually one and then no naps (with longer consolidated sleep at night). Furthermore, infant sleep is characterized by larger amounts of REM sleep, suggesting that REM sleep may have important consequences for the developing brain [76]. Infant sleep is important to the evolutionary story of sleep in two other ways: one involves the role of infant-parent co-sleeping, and the other involves infant crying.

Infant-parent co-sleeping has attracted much attention in recent decades, with parents faced with the dilemma of sleeping with the baby versus putting the baby in a separate room. All discussions of co-sleeping should begin by appreciating how radically novel it is for dependent children to even have the option to sleep separately from their caregivers. Throughout evolutionary history, families slept together, possibly with extended family members, and the same is true in many traditional societies today [59, 77]. It is only in modern living conditions—with increased safety and availability of separate bedrooms for parents and children—that the dilemma of infant–parent co-sleeping arises.

James McKenna was among the first anthropologists to investigate mother–infant night-time interactions empirically, often injecting an evolutionary perspective [78, 79]. In some of this research, the investigators found that bed-sharing resulted in less deep sleep for mothers and infants, but more simultaneous awakenings by mothers and infants that were associated with more breastfeeding [24]. Thus, mothers would tend to awaken or transition between sleep states at times when babies were also likely to awaken, resulting in less disruption to the mothers’ sleep cycles and a higher feeding frequency for infants [80]. Overall, these studies demonstrate a mutually reinforcing relationship between mother–infant co-sleeping and feeding, probably reflecting correlated evolution among these behaviors.

This research has been used to inform the potential risks associated with solitary sleep practices; e.g. the lack of breastfeeding and solitary sleeping has been identified as a risk factor for sudden infant death syndrome (SIDS), suggesting that less deep sleep in infants who were co-sleeping and breastfeeding more regularly were at lower risk of SIDS [81, 82]. However, other studies have found that bed sharing also increases risk of SIDS, which may be amplified by factors such as infant age or use of alcohol or drugs [83].

The other insight to infant sleep comes in the context of infant crying, a feature not observed in chimpanzees [84]. Haig [23] revived and extended a hypothesis [85] that night-time arousal and crying by infants is an adaptive behavior to extend inter-birth intervals, benefiting the crying infant at the potential cost to parental reproductive success. Reviewing the literature, Haig [23] notes that shorter inter-birth intervals lead to greater offspring mortality, and that more night-time breastfeeding episodes results in longer postpartum amenorrhea. Thus, ‘natural selection will have preserved suckling and sleeping behaviors of infants that suppress ovarian function in mothers because infants have benefited from delay of the next birth’ (p. 34). Additionally, Haig [23] incorporated modern perspectives of genomic conflict by considering how imprinted genes of maternal origin might favor more consolidated sleep, whereas genes of paternal origin promote greater wakefulness.

As noted by Haig [23], the explicit inter-generational and intra-genomic conflicts in his proposal challenge the assumption of mother–infant co-sleeping as a highly co-evolved and harmonious system that was suggested above in some of the research on co-sleeping. Instead, Haig’s [23] research suggests a need to appreciate that substantial parent-offspring conflict likely exists even in the context of sleep.

## MAMMALIAN SLEEP IN COMPARATIVE AND THEORETICAL PERSPECTIVE

To understand the reasons for short human sleep discussed above (Fig. 1), we can turn to comparative variation in mammalian sleep to ask, ‘what are the factors that influence sleep durations across species?’ Are these factors related to the function of sleep, for example involving the brain, or circadian release of growth hormone? Or are ecological factors more informative of sleep durations, perhaps because they constrain how much time is available for sleep? This comparative perspective can help uncover the factors that have led humans to sleep so differently from other primates (and perhaps more similarly to other mammals). Although many earlier studies have investigated comparative patterns of sleep [86–88], here we focus on more recent studies that made use of larger sample sizes and improved statistical-phylogenetic methods [63].

Two independent research groups [89, 90] have investigated the phylogenetic, ecological and life history drivers of sleep architecture, which is defined as the quantitative structure and pattern of sleep. Sleep architecture includes variables related to total sleep time, duration of REM and NREM sleep, duration of the NREM–REM cycle, and distribution of sleep through the 24-h period into one or many bouts (i.e. monophasic vs polyphasic, respectively). We consider the major hypotheses for sleep duration that have been investigated comparatively, which fall into two broad categories: those in which ecological factors, such as diet, influence sleep durations; and other hypotheses proposing that specific functional benefits of sleep, such as memory consolidation, influence sleep architecture. Among the ecological factors, several variables are considered to be important: (i) predation risk, with longer sleep times expected when animals have access to a safe and stable sleep site; (ii) metabolism, with higher metabolism either favoring more sleep to conserve energy, or less sleep to enable animals to better meet nutritional needs; and (iii) body mass (or its correlates), with larger bodied animals needing more resources and thus having less time for sleep. In terms of functional benefits of sleep, one major hypothesis involves memory consolidation, with larger brained animals proposed to need more sleep [91]. Another functional benefit involves immune function, with animals exposed to more parasites and pathogens allocating more time to sleep and use of that “down-time” to reallocate energy to increase the standing crops of disease-fighting leukocytes (and other potential mechanisms of improved immune defenses).

Lesku *et al.* [89, 92] and Capellini *et al.* [90] applied new phylogenetic methods to investigate the evolution of mammalian sleep, working independently and with somewhat different methods and approaches. From these studies, we can draw several conclusions. First, predation risk appears to be a major predictor of sleep architecture in mammals, with safer options for sleep leading to more sleep. Similarly, animals at lower trophic levels (e.g. herbivores) sleep less than those at higher trophic levels (e.g. carnivores). Second, relative brain mass shows no association with sleep durations, but does covary positively with the percentage of REM sleep across mammals; specific brain regions were also generally unrelated to sleep architecture, with the exception of the amygdala and NREM sleep [91]. Third, basal metabolic rate (with and without control for body mass) showed a negative association with sleep durations, suggesting that greater metabolic needs in a lineage favors less sleep. Fourth, animals with longer gestation lengths sleep less, even after controlling for body mass. Fifth, species with more sleep have higher white blood cell counts and possibly fewer parasites [93]. Finally, the durations of REM and NREM sleep covary positively [94]. This finding suggests that the total amount of time spent in different sleep stages does not strictly reflect specific functional benefits associated with those states; instead, animals add both REM and NREM when ecological conditions provide opportunity for more sleep.

Overall, results from these studies suggest that ecology is the primary driver of sleep durations, with evolution adjusting sleep durations across species based on the benefits and costs of being awake for predation, foraging and social interactions. In other words, tradeoffs between sleep and other activities are more central to understanding comparative variation in sleep, and more so than functional benefits of sleep. Functional benefits may instead be acquired through deeper sleep during particular sleep stages [95]. This tradeoff perspective is highly relevant to understanding the short duration of human sleep: it suggests that if an animal has something better to do than sleep (such as forage, court potential mates or watch for predators), natural selection will favor shorter sleep durations.

Based on these findings across mammals, we argue that activities that are crucial for success in humans—such as learning new knowledge or skills, and building and cementing social bonds through social activity—are so important for reproductive success that natural selection has favored the expansion of these activities beyond daylight hours [21], despite the probable costs for cognitive, metabolic and immune function. Sleeping on the ground likely also increased risk of predation and potential for attacks by hostile conspecifics, favoring less sleep. Although we hear many sleep scientists and doctors lament the temptations of digital media as counter to healthy sleep, the phylogenetic comparative results from above suggest that natural selection has been hard at work eroding human sleep for many millennia. We therefore expect to find that across societies, humans run sleep debts, even among hunter-gatherers and traditional agriculturalists. In support of this, recent work with actigraphy devices in three pre-industrial populations revealed that hunter-gatherers do not sleep more than ‘modern’ humans, with average sleep durations of only 6.5 h per night [69]. Similarly, research on rural Haitian [95] and Malagasy agriculturalists [96] without access to electricity found that they also sleep only 6.5–7 h per night on average.

## EVOLUTIONARY PERSPECTIVES ON SLEEP DISORDERS

Evolutionary perspectives can shine new light on human health, in many cases providing new treatment options while also enhancing our understanding of the underlying causes of these disorders [97, 98]. Sleep biologists have engaged with evolutionary perspectives for decades [86, 87, 99, 100], and some studies from within and outside sleep medicine have applied an evolutionary perspective to investigate human sleep disorders [22, 59, 64, 101]. We consider some of these attempts.

### Insomnia

Insomnia is defined as persistent difficulty falling or staying asleep despite the adequate opportunity to do so, and is associated with significant impairment in function or reduced quality of life; it has a population prevalence of ∼10% [102, 103]. Multiple lines of evidence suggest that insomnia is generally associated with a state of hyper-arousal, which includes multiple alterations involving activation of the sympathetic nervous system and diminished homeostatic drive for sleep [104, 105].

Consistent with this framework, McNamara and Auerbach [101] considered insomnia to result from stress and hyper-vigilance associated with some external threat. In essence, they viewed insomnia as an adaptive trait under circumstances of perceived threat. In support of the view that sleep need can be adjusted based on perceived threats, research on insomniacs has revealed relatively less daytime sleepiness and lower cognitive costs of sleep deprivation, when compared with the adverse consequences of depriving those without insomnia of sleep [104, 106]. This suggests that individuals may sacrifice a small decline in cognitive function for overall broader vigilance against a perceived threat. People diagnosed with insomnia may complain of daytime impairment (indeed, it is included in diagnosis), yet natural selection operates on the reproductive benefits conferred by facilitating survival of self and kin.

Taking an evolutionary perspective, it makes good sense for mechanisms to evolve that suppress the need to sleep when threats exist, such as the presence of predators or conspecific competitors. In today’s society, however, these threats are substantially less common (although they may persist in dangerous neighborhoods in developed countries or in the growing urban populations of developing countries, see below and reference 106). Loss of sleep due to anxiety before an examination or other stressful event is hardly as useful as it might have been when vigilance was needed for physical threats. Thus, we have a mismatch situation in which potentially adaptive solutions from our ancestral past are no longer beneficial to many people living today in safe environments (which is an evolutionary novelty). From a clinical perspective, this strongly suggests that doctors need to alleviate the sources of the anxiety and stress to effectively treat insomnia [101], or to teach effective coping strategies when the perceived threats are unresolvable. For patients, understanding evolutionary drivers of this disorder—at least when dysfunction is associated with some stressor—may also help individuals overcome insomnia.

Middle of night insomnia may represent a different situation. As noted earlier, the historical record in Europe indicates that many populations exhibited a biphasic sleep pattern: a ‘first sleep’ that was interrupted by middle-of-the night activity, followed by a ‘second sleep’ [64]. From this, it is reasonable to hypothesize that middle of night insomnia is a relic of a long-term, evolutionarily adaptive sleeping pattern. If biphasic sleep patterns were under selection in populations that have experienced highly seasonal fluctuations in day length (i.e. at high latitudes), one would expect that middle-of-the night insomnia characterizes individuals with ancestors from these regions, perhaps for adaptive reasons such as ensuring one’s family is warm and well fed during long winter nights.

An alternative viewpoint should also be considered. Middle of the night ‘awakening’ should be differentiated from middle of the night ‘insomnia’; the former may be normal, while the latter pathological and representative of underlying problems with maintaining sleep. The hallmark of the non-pathological phenomenon is the absence of daytime impairment. Those with middle of the night awakening and difficulty returning to sleep have greater tendency towards impairment and complaint than those with sleep onset problems [108, 109]. Some experts have proposed that sleep onset problems reflect different underlying causes than middle of the night insomnia; for example, sleep onset problems may reflect stress, effects of light at night or delays of circadian phase, whereas middle-of-night insomnia may reflect maintenance problems and a true inability to sleep given an adequate opportunity to do so [108–110].

### Narcolepsy

Narcolepsy presents another interesting situation from an evolutionary perspective, again with a mismatch or ‘novel environment’ component to its etiology. Narcolepsy afflicts about 0.02–0.03% of the US population [111]. This low prevalence suggests that narcolepsy itself is not adaptive, and that instead, we need to consider how evolution has made humans susceptible to this disease. Individuals with this condition experience excessive daytime sleepiness; a majority suffer from cataplexy (i.e. where an emotionally salient event can trigger an intrusion of paralysis), such as occurs typically during REM sleep, while the affected individual remains conscious. Narcolepsy often first appears in adolescence. Evidence suggests that the condition likely involves an autoimmune process, with symptoms of the disorder arising as a consequence of autoimmune destruction of the hypocretin (orexin) neurons in the hypothalamus [111].

In this context, narcolepsy presents two evolutionary angles to explore. First, what accounts for the genetic variants that lead to this condition, especially involving potentially adaptive consequences of narcolepsy-related genetic variants in past or present environments? Second, does modern life involve new environmental factors that trigger the onset of narcolepsy in those with genetic variants associated with narcolepsy? We proceed to each of these in turn.

Several studies have identified genetic variants associated with narcolepsy, including variants in the human leukocyte antigen loci that are involved in immune responses [112]. For example, one recent genome-wide association study identified a genetic variant in the purinergic receptor subtype P2Y11 gene that is associated with narcolepsy [113]. This variant is involved in substantially reduced expression of the gene in natural killer cells and CD8+ T-cells, and reduced resistance to apoptosis in these cells.

Although these genetic studies may explain the link to narcolepsy and potentially other autoimmune diseases, it is unclear whether the alleles were favored by natural selection. If some genetic variants show tradeoffs arising from antagonistic pleiotropy, for example, we might expect some other phenotypic benefits that outweigh the potential costs of narcolepsy in a subset of individuals. Alternatively, it could be that narcolepsy and the associated genes reflect ancient adaptations that are no longer relevant, and have been carried along due to low selective pressure against them. One such explanation views genes associated with narcolepsy as evolutionary hangovers (atavisms) that originally had a role in predator defense. In particular, the genes may have been adaptive in the context of feigning death as a last resort to predator attacks (i.e. tonic immobility, a widespread response to predators among vertebrates and invertebrates). According to one recent hypothesis, REM sleep—with its associated paralysis—may have its roots in tonic immobility [114]. Researchers have identified neurological similarities between the paralysis in narcolepsy and tonic immobility in animals [115].

A second perspective invokes evolutionary mismatch. In addition to genes, it appears that environmental triggers are important in narcolepsy. Evidence for this view includes discordance between genetic variants and disease across populations [116], and low concordance of narcolepsy among monozygotic twins and other family members [117]. In addition, narcolepsy is best documented in humans and in domesticated animals that have close and regular contact with humans, such as dogs, horses and sheep [see 118]. This concordance is consistent with all these animals and humans experiencing a common environmental factor in modern environments. Hence, the search has been on for environmental factors that might cause narcolepsy in those with genetic backgrounds that make them susceptible.

Infectious agents have been among the factors thought to act as an environmental trigger for narcolepsy [111]. Potential links to H1N1 influenza have received the most attention, including potential triggers by influenza vaccines. Following the vaccination campaign against the H1N1 epidemic in 2009–10, increased cases of narcolepsy were reported in Europe [116]. Similarly, increased incidence of narcolepsy was reported in China after the H1N1 epidemic, and onset was found to be seasonal in other years, occurring at higher rates after the cold-and-flu season [119]. However, the findings in China are unlikely to be linked to vaccinations. In addition to influenza, high levels of antibodies (anti-streptolysin O) to *Streptococcus pyrogenes*—causal agent of strep throat—have been linked to narcolepsy [117].

Overall, we propose that narcolepsy represents a disorder that has ancient genetic roots, with some genetic variants having insufficient environmental triggers to express themselves until the present. When these genetic variants were expressed throughout our evolutionary history, they may have been under low levels of selection for their removal from the population, and that is even truer today. Whether these genetic variants have fitness benefits remains unknown, but it seems unlikely given the extremely low prevalence. The well-documented cases of narcolepsy in domesticated animals, such as sheep and dogs, also exemplifies the benefits of investigating this question in a One Health framework [120], which considers how the health of humans, animals and the environment are interconnected. Future research may find, for example, that a common environmental trigger influences disease in all these species.

### Circadian rhythm disorders

Disorders of circadian rhythm are defined by a mismatch of an individual’s natural sleep period and the desired sleep period based on the social environment [121]. Circadian rhythm disorders are characterized by various types of sleep period mismatch, such as: ‘delayed sleep phase syndrome’, in which affected individuals tend to go to bed later and sleep later than ideal for optimal function in their environment; ‘advanced sleep phase syndrome’, in which affected individuals tend to fall asleep earlier and wake up earlier than preferred; and ‘irregular sleep wake schedule’, where a shifting mismatch occurs between the period in which an individual is able to sleep and their preferred sleep period. The population prevalence of these conditions varies, with the most prevalent being delayed sleep phase syndrome, which has a prevalence of 7–16% among adolescents [121].

These conditions have a genetic basis that is modulated by developmental and environmental factors [121]. They actually only represent disorders in the sense that society demands a particular sleep–wake schedule that cannot be met without the development of symptoms by individuals who deviate from the norm, but who are really ‘normal variants’ in that they are asymptomatic if allowed to sleep on their preferred schedule. Yet, it could be argued that variant sleep–wake patterns are actually beneficial for many individuals and societies, allowing them to serve necessary roles for society. This includes night workers, shift workers, and those who need to work for extended shifts before sleep is possible.

From an evolutionary perspective, benefits may have accrued to those with slightly different circadian cycles—or different ‘chronotypes’—with benefits for their communities, too. Just as there exist multiple roles in today’s society to meet demands of economic growth and around-the-clock safety, having individuals in a social group on different sleep schedules may have been beneficial in our evolutionary past, even in hunter-gatherer populations. Indeed, a hunter-gatherer community in which at least one person is always vigilant would presumably be better protected from hostile conspecifics or predators. Variability in chronotype is heritable in humans and has been shown to differentially affect reproductive output [122, 123].

Treatments exist to help affected individuals normalize their sleep–wake schedules to a modest degree and to diminish the symptoms associated with circadian rhythm disorders. For most individuals, however, the best strategy may be to guide them towards lifestyles that best match their natural circadian proclivities. For those in school, this may include a late start to the school day or permission to nap. Individuals with delayed sleep phase syndrome will generally function best in jobs that can start in the afternoon. Those with irregular sleep/wake schedules often do best in self-employment situations or loosely structured jobs. Finally, patients may take solace in understanding that phenotypic variation in sleep phasing may reflect adaptive strategies in ancestral environments.

### Seasonal affective disorder

Seasonal affective disorder (SAD) is characterized by the presence of symptoms of depression that recur every winter and remit every summer [124]. Many affected individuals also report a mild hypomania during the spring and summer. The pathophysiology of this condition appears to lie in a deviant response to decreased exposure to light in the winter. As would be expected on this basis, SAD is more common in extreme latitudes with very short day lengths during the winter. For example, the prevalence of SAD is <1% in the USA, but 2–3% in Canada [124]. The prevalence is higher in women than men, and tends to occur during childbearing years in women [125].

Some evidence suggests that SAD is actually a circadian rhythm disorder because melatonin production, which is normally suppressed by light exposure, is increased in extent and duration in those suffering from SAD compared with their neighbors without SAD [124]. On this basis, it is hypothesized that individuals with SAD experience a shift in their circadian sleep–wake schedules that makes them more lethargic during the day, particularly on winter mornings. This explanation is also consistent with the phenotype of SAD, which in contrast to unipolar major depression, is more likely to be associated with reports of daytime sleepiness and lethargy. Given the presumed pathophysiology, it is not surprising that the treatment of choice for this condition is light exposure therapy [124].

It has been hypothesized that SAD is adaptive in highly seasonal environments, where increasing sleep during the winter would conserve energy and maintain thermoregulation, while increased energy and capacity for work would be beneficial during warmer, more productive months, coupled with potential advantages of SAD symptoms for pregnant women during the winter months [126, 127]. It is easy to appreciate the adaptive value of such a trait prior to electrical lighting and in agrarian societies, and in areas with long, dark winters and food production concentrated in a subset of the year. If future research finds support for this hypothesis, SAD would be adaptive for a relatively small (and shrinking) percentage of the world that lives at high latitudes without access to modern lighting, and thus could be considered to be an evolutionary mismatch condition [126, 127].

### Sleep-disordered breathing (sleep apnea)

We close this section by considering sleep disordered breathing, which involves a wide range of breathing abnormalities during sleep. We focus especially on obstructive sleep apnea. This form of sleep apnea occurs when the tone in muscles that support the upper airway decreases during sleep to the point that they are unable to prevent the forces impinging on the airway from causing a collapse, which then obstructs the airway (i.e. ‘apneas’). With obstructive sleep apnea, breathing can be blocked multiple times per hour, resulting in gasping, awakening and reduced blood oxygenation. It is differentiated from central sleep apnea, which involves a central nervous system-mediated absence of effort to breathe [128].

Risk factors for obstructive sleep apnea include obesity, large neck circumference, use of alcohol before bedtime, and smoking, yet genetic and anatomical features are also important, including characteristics of the airway [129]. Thus, obstructive sleep apnea may be seen as an example of an evolutionary mismatch disease, with over-abundant access to high calorie food, distilled alcohol, a sedentary lifestyle and tobacco fueling the rise in this condition, especially at older, post-reproductive ages. However, the genetic-anatomical features may be maintained for reasons that are not yet clear, and result in increased risk of sleep apnea later in life, potentially independent of behavioral risk factors. Although most patients diagnosed with obstructive sleep apnea opt for medical treatment, prevention through a healthy diet and exercise may be an option for some patients.

## GLOBAL HEALTH, SLEEP AND EVOLUTIONARY MEDICINE

Many factors are changing sleep patterns and sleep quality globally, including expanded use of artificial lighting, shift-work, use of screen-based digital media and excessive environmental stimuli in urban environments. Sleep is a critically important aspect of health; as noted earlier, it is intimately connected with almost every aspect of human health, including immune function, metabolism, and cardiovascular disease. Sleep is also critical for effective working memory, attention, visual-motor performance, and decision-making, with disrupted or irregular sleep resulting in declines in workplace productivity and increases in accidents [e.g. 9, 129, 130, 131]. Despite these strong links between sleep and health—and despite the pervasive changes in sleep in developed countries—few studies have considered the health implications of chronic sleep deprivation in a global health context [11].

Perspectives from evolutionary medicine are important for understanding global health challenges associated with changing sleep patterns. These perspectives include the concept of evolutionary mismatch, where changes in environments and lifestyles today differ from those in our ancestral past in ways that create new health problems. Potential sources of mismatch include: more widespread use of electrical lighting and new social connectivity enabled by technology; populations that live at exceptionally high densities, resulting in sleep disruptions due to noise and perceived risks at night; changes in other dimensions of health that may impact sleep, such as rising rates of obesity; and changes in diverse sleep practices involving mother–infant co-sleeping, ambient light and poor quality bedding that may affect musculoskeletal health. Evolutionary medicine perspectives also aim to understand adaptations for adjusting sleep in times of need, such as sleeping less to monitor the environment in risky settings [107], or tradeoffs between sleep and other fitness (or financial) enhancing behaviors (e.g. as shown by extreme sleep deprivation in male pectoral sandpipers during the mating season [133]). Finally, evolutionary medicine is important for understanding sleep disorders, which may increase globally as more populations adopt Western lifestyles in terms of diet, lighting and night-time entertainment.

Health disparities are starting to be linked to sleep disparities and their drivers, and a multi-disciplinary group of sleep biologists, public health specialists, economists and anthropologists are investigating the ways that sleep, health, ethnicity and socioeconomic status are intertwined [134–137]. In one study, for example, Hale and Do [138] found that compared to white Americans, African Americans, Hispanics, and non-Hispanic ‘others’ showed a higher rate of ‘short’ (≤6 h) sleep, which is known to be associated with poor health outcomes. They also found that living in an inner city was associated with increased risk of short sleeping, suggesting that some of these ‘sleep disparities’ [137] reflect stress and noise associated with living in highly urban, socioeconomically disadvantaged neighborhoods. Another study of Americans found that perceived racial discrimination covaried with sleep disturbance [139, see also 140]. An important area for the future is to apply these perspectives to health in developing countries, particularly in the growing urban environments that represent significant sources of noise, stress and risk, often with inadequate places for sleep.

Major efforts are afoot to expand access to electricity for low- and middle-income countries, e.g. through America’s USAID’s ‘Power Africa’ initiative (https://www.usaid.gov/powerafrica; accessed 19 July 2016). Greater use of electric lighting will be one outcome, which we expect will lead to later bedtimes as people make greater use of the day for work, education and socializing [141]. Similarly, television and other forms of entertainment and communication may distract people from healthy sleep practices. Collectively, we expect that these developments will lead to a greater sleep debt in developing countries, and will thus contribute to rising rates of obesity, heart disease, diabetes and other non-communicable diseases in these countries. These effects will be especially acute when coupled with increased access to Western diets and lifestyles. Many of these countries will continue to have a high infectious disease burden, especially near the equator. Evidence points to increased risk of infection if sleep times decline [16, 17], including comparative results noted earlier [93]. Thus, it is critical to evaluate the ways that westernization impacts sleep patterns, and the health effects of these changes in different populations.

## CONCLUSIONS

We are finally beginning to make sense of why we sleep, and to understand the origins of some of the interesting sleep adaptations across the animal kingdom, such as unihemispheric sleep in aquatic mammals as an adaptation for obtaining oxygen [142, 143]. It appears that human sleep architecture differs from our closest living relatives, with humans packing a higher percentage of REM sleep into shorter total daily sleep durations. The large evolutionary changes in sleep along the human lineage may be responsible for some sleep disorders that are seen in humans, although even some of the most striking disorders, such as narcolepsy, also occur in other animals [118]. The rapid transformations in sleep that are occurring globally today should be of great concern, yet scientists are only just beginning to investigate the implications of sleep for health disparities in both developing and developed countries. Concepts from evolutionary medicine—such as mismatch, tradeoffs, and adaptation to local environmental settings—are important for understanding these transformations. An interdisciplinary perspective will be essential in this endeavor.

Studying sleep requires a comparative approach, as emphasized throughout this review. Comparative approaches are needed to investigate the adaptive function of sleep and the factors that constrain sleep. Such approaches also contribute to understanding the sleep disorders that plague a remarkably high percentage of people around the world. In this context, it is critically important to obtain comparable data on sleep in different populations, with the aims of better understanding the genetic underpinnings of sleep, phenotypic plasticity in sleep, and the potential for adaptive differences in sleep architecture in different populations. Although many adaptive hypotheses can be forwarded for aspects of sleep disorders (as reviewed earlier), we expect that most of these patterns are not shaped by natural selection as adaptations, but rather reflect weak selection against them in our evolutionary past, with resulting variation in the population. Alternatively, it may be that natural selection has shaped other aspects of biology—such as human metabolism, cognition and development—in ways that now make us vulnerable to sleep disorders, especially in the stress of modern environments. An understanding of the evolutionary basis for sleep disorders may bring some consolation to those who face anxieties about their sleep and could guide better outcomes in a globalizing and rapidly developing world.
